# Disentangling Gender and Relative Age Effects in Women’s and Girls’ Rugby Union

**DOI:** 10.3390/jfmk9020061

**Published:** 2024-03-29

**Authors:** Adam L. Kelly, Diogo Coutinho, John M. Radnor, Kate Burke, Donald Barrell, Daniel Jackson, Paolo R. Brustio

**Affiliations:** 1 Research for Athlete and Youth Sport Development (RAYSD) Lab, Research Centre for Life and Sport Sciences (CLaSS), College of Life Sciences, Faculty of Health, Education, and Life Sciences, Birmingham City University, Birmingham B15 3TN, UK; daniel.jackson6@mail.bcu.ac.uk; 2 Department of Physical Education and Sports Sciences, University of Maia (UMAIA), 4475-690 Maia, Portugal; diogoamcoutinho@gmail.com; 3 Department of Sports Sciences, Exercise and Health, University of Trás-os-Montes and Alto Douro, 5000-801 Vila Real, Portugal; 4 CreativeLab Research Community, Research Center in Sports Sciences, Health Sciences and Human Development, CIDESD, 5000-801 Vila Real, Portugal; 5 Youth Physical Development Centre, School of Sport and Health Sciences, Cardiff Metropolitan University, Cardiff CF23 6XD, UK; jradnor@cardiffmet.ac.uk; 6 Rugby Football Union, Rugby House, Twickenham Stadium, London TW2 7BA, UK; kateburke@rfu.com (K.B.); donaldbarrell@rfu.com (D.B.); 7 Department of Clinical and Biological Sciences, University of Turin, 10126 Turin, Italy; paoloriccardo.brustio@unito.it; 8 NeuroMuscularFunction Research Group, School of Exercise & Sport Sciences, University of Turin, 10126 Turin, Italy

**Keywords:** talent identification, talent development, athlete development, age grouping, youth rugby, rugby football union, female rugby, female sport

## Abstract

Relative age effects (RAEs) within sports refer to the overrepresentation of athletes born earlier in the selection year and the underrepresentation of those born later in the selection year. Research examining RAEs in women’s and girls’ rugby union remains limited in comparison to the male literature, whilst the impacts of RAEs on the youth–senior transition are yet to be explored in a female sport context. As such, the purpose of this study was to examine RAEs during entry into the women’s and girls’ premiership and international rugby union pathways in England, as well as to compare them to their respective senior cohort (*n* = 1367): (a) U18 England Rugby Centre of Excellence Player (*n* = 325) vs. Senior Premiership Player (*n* = 868), and (b) U18 England Player (*n* = 49) vs. Senior England Player (*n* = 125). Chi-square (χ^2^) analyses compared birth quarter (BQ) distributions against expected distributions. The findings revealed no significant difference in BQ distributions at either youth or senior levels, as well as no significant differences in the BQ distributions of those who were likely to transition from youth to senior levels (all *p* > 0.05). Importantly, though, descriptive statistics showed a skewed birthdate distribution in both U18 England Rugby Centre of Excellence Player (BQ1 = 30% vs. BQ4 = 20%) and U18 England Player cohorts (BQ1 = 33% vs. BQ4 = 18%). We highlight the gender-specific mechanisms that potentially explain the variations between male and female RAEs in rugby union, including developmental differences, sport popularity, and sociocultural norms. We also warn against a ‘copy and paste’ template from the male provision to ensure the recent growth of female rugby union does not fall victim to the same RAEs in the future.

## 1. Introduction

In an attempt to create equitable competition in youth rugby union, a common practice is to group players into annual age categories using fixed cut-off dates [1]. Inadvertently, however, these organizational structures generally offer relatively older youth (i.e., those born near the start of the selection cut-off date) a variety of developmental advantages over their relatively younger peers (i.e., those born towards the end of the selection cut-off date), termed ‘relative age effects’ (RAEs). More specifically, RAEs refer to the participation, selection, and attainment inequalities in the immediate, short-term, and long-term across sports due to this chronological age group approach, which is a well-known phenomenon in men’s and boys’ youth rugby union (see [2] for a review). Often, players born earlier in the start of the section's cut-off date possess higher physical (e.g., body height, strength), technical (e.g., skill development resulting from a higher experience), tactical (e.g., ability to perceive and interact with available space), and psychological (e.g., higher confidence) attributes than their younger counterparts (e.g., [3,4,5]). Such RAEs are present at recreational/age-grade (birth quarter one (BQ1) = 29% vs. BQ4 = 22%; [6]), high school (BQ = 39% vs. BQ4 = 13%; [7]), regional (BQ1 = 46% vs. BQ4 = 14%; [8]), academy (BQ1 = 42% vs. BQ4 = 8%; [9]), and youth international (BQ1 = 40% vs. BQ4 = 18%; [10]) levels. Contextual factors, such as age (e.g., U7 vs. U18; [11]), competition level (e.g., amateur vs. professional; [12]), nationality (e.g., northern vs. southern hemisphere; [13]), and position (e.g., forwards vs. backs; [14]), appear to play an important role relative to the extent of RAE occurrence in the male rugby union.

Although RAEs are consistently prevalent at boys’ youth levels, findings are mixed at men’s senior levels. For example, Kelly and colleagues [4] showed that there were no RAEs in professional (BQ1 = 27% vs. BQ4 = 24%) or international (BQ1 = 25% vs. BQ4 = 27%) male rugby unions in England, despite displaying RAEs across all youth cohorts. In contrast, RAEs have been revealed across rugby union hotspots, such as Australia [15], France [16], Italy [17], and New Zealand [12]. When playing position was added to the equation, Kearney [18] demonstrated how RAEs were influenced by position in both professional and amateur French populations, whereby it existed for forwards but not backs (BQ1 = 26% vs. BQ4 = 20%). Moreover, Kearney [19] adopted a cross-cultural comparison of four popular rugby union nations (i.e., Australia, England, New Zealand, and South Africa), revealing that only South Africa had pronounced RAEs across all playing positions at the senior level (BQ1 = 31% vs. BQ4 = 21%). Together, these studies suggest differences in both position and national sport culture may be important considerations when exploring who is at risk of RAEs. These findings also imply that RAEs may be considerably less prominent at senior levels compared to youth levels in men’s and boys’ rugby union.

There appears to be a complicated relationship between selection at youth levels and the successful transition to senior levels in male rugby union. Initial findings from McCarthy and colleagues [9,20] identified ‘reversal effects’ of relative age when exploring the transition from the academy to a professional level at an English Rugby Premiership club. As an example, McCarthy and colleagues [20] revealed that although there were RAEs at the academy level favouring relatively older players (BQ1 = 41% vs. BQ4 = 16%), there was a greater proportion of relatively younger players who successfully converted to the senior professional level (BQ1 = 2% vs. BQ4 = 11%). Caution should be taken, however, when considering the proportion and absolute values, as there may still be more relatively older players successfully transitioning. Kelly and colleagues [21] also showed that whilst there was a significant overrepresentation of relatively older U15 regional academy (BQ1 = 43% vs. BQ4 = 10%) and U16–23 England academy (BQ1 = 37% vs. BQ4 = 15%) players, when exploring the likelihood to transition to their respective senior cohort, BQ4s were 3.9 times more likely to achieve senior professional and senior international levels than BQ1s and BQ2s, respectively. Despite the aforesaid male results, to our understanding, there is yet to be a study that has determined the youth–senior transition in female rugby union, with a recent study from our research group being the first of its kind in the context of football [22].

In comparison to the male relative age research, little is known about their female equivalents. To our knowledge, there are only two studies that have examined RAEs in the female rugby union [11,23]. First, Lemez and colleagues [23] evaluated the BQ distributions in the New Zealand (ages 4 to 21 years; *n* = 13,899) and Canadian (ages 4 to 21 years; *n* = 1497) developmental leagues alongside senior World Cup tournaments (2006 and 2010 rosters; *n* = 498). They found mixed results with little presence of RAEs within their New Zealand (BQ1 = 25% vs. BQ4 = 24%), Canadian (BQ1 = 27% vs. BQ4 = 21%), and World Cup (BQ1 = 23% vs. BQ4 = 27%) samples. Second, Kelly and colleagues [11] explored the English age-grade rugby union participation trends from U7 to U18 (*n* = 23,563), revealing significant RAEs across the majority of annual age categories (overall: BQ1 = 28% vs. BQ4 = 23%). Interestingly, however, further analysis revealed an inverse RAE in the triennial U18 age group (i.e., U16, U17, and U18s were banded in a single 36-month age group), whereby those born in the first quartile of the age group (i.e., those born in the first 9 months of the selection age group: 18%) were underrepresented compared to those born in the fourth quartile of the age group (i.e., those born in the first 9 months of the selection age group: 30%). In comparison to the male relative age literature, these preliminary studies demonstrate there are likely different effects in female rugby unions.

England Rugby has developed its own action plan, *Every Rose 2021–2027 Action Plan* [24] (p. 1), which aims to build on the successful growth of women’s and girls’ rugby by increasing opportunities for them to play the sport. The plan defines itself as being about ensuring the sport has “the right infrastructure and support in place, from grassroots to performance, to accelerate growth and create long-term value”, and the strategy aims to make women’s and girls’ rugby “accessible, successful, visible, and commercially viable”. According to official statistics from England Rugby from a recent press briefing (e.g., [25]), there was a 25% increase in the number of women (aged 18+ years) registered to play rugby union following the COVID-19 pandemic, which means that there are now more women playing rugby union than ever before. Specifically, over the past five years, the number of women playing rugby union in England has grown from ~25,000 to ~40,000 [25], with England Rugby targeting to grow the numbers to 100,000 by 2027 as part of its strategy. The number of girls’ teams at clubs has increased in all age categories in the past two years as well, with U18s up 111% to 360, U15s up 77% to 421, and U13s up 10% to 385 [25]. Additionally, due to the growth in popularity, there are now enough girls playing rugby in England to be able to introduce the additional age band at U12 and reduce the U18 triennial age band to a dual band, which previous findings showed could have created unintended consequences on participation and dropout [11]. Therefore, it will be important to monitor these changes and strategies to evaluate whether they are achieving their aims whilst ensuring they are not having unintended consequences, such as creating RAEs in women’s and girls’ talent pathways.

Recent reviews, book chapters, and empirical studies have underscored how female populations remain underrepresented throughout talent identification and development research. In light of this, and given the lack of research specifically in female rugby unions, it is important to gain a better understanding of women’s and girls’ RAEs in order to capture an even-handed picture of how they occur. Based on current relative age research in youth sport, male RAEs appear to be more pronounced, whereas female RAEs seem to be more inconsistent [26]. Researchers have explained this could be a result of contextual factors such as physiological differences, sport popularity, allocated funding, and youth sport policies [2]. Due to the gender-specific relative age mechanisms between the two sexes, it is essential to recognise that findings from many male studies may not be transferable to female cohorts; thus, research is needed to better understand RAEs specifically in women’s and girls’ rugby union. As such, the purpose of this study was to (a) explore the BQ distributions of the premiership and international player pathway in England and (b) examine the likelihood of achieving senior premiership and international status once selected at the youth level. Based on the existing relative age literature within rugby union, we hypothesized that (a) there would be RAEs across youth cohorts but not in senior cohorts, and (b) there would be a greater proportion of relatively younger players who achieve the senior professional and international status once selected at youth levels.

## 2. Materials and Methods

### 2.1. Sample

The women’s and girls’ rugby union talent pathway in England is quickly evolving. Currently, the first step towards a senior Premiership club is through an England Rugby Centre of Excellence that competes in a U18 age group (with ages ranging from 15 to 18 years), whilst the initial entry into the national programme is through England U18s (with ages ranging from 17 to 18 years). To capture this pathway, the current study allocated each player (total *n* = 1367) into one of the four cohorts based on their playing level: (a) U18 England Rugby Centre of Excellence Player (*n* = 325; every registered player during the 2020/21 season), (b) U18 England Player (*n* = 49; every registered player during the 2020/21 season), (c) Senior Premiership Player (*n* = 868; every registered player from 2017/18 to 2020/21), and (d) Senior England Player (*n* = 125; every player who achieved a minimum of one cap from 1986/87 to 2019/20). The dataset was provided by England Rugby, which included all the retrospective data they had available with any duplicates removed. There were 18 individuals in the U18 England Rugby Centre of Excellence Player cohort and 105 individuals in the Senior England Player cohort whose birthdate data were unavailable, and therefore, were not included in this study. There were 18 individuals in the U18 England Rugby Centre of Excellence Player cohort and 105 individuals in the Senior England Player cohort whose birthdate data were unavailable, and therefore, were not included in this study. This study was authorized by England Rugby and gained ethical approval from the Health, Education and Life Sciences Faculty Academic Ethics Committee at Birmingham City University (reference code: Kelly/6263/R(V)/2020/Mar/HELS FAEC).

### 2.2. Measures

In accordance with English annual-age group cut-off dates in rugby unions, the year was divided into four three-month BQs, starting with September 1st as ‘month 1′ and ending with 31 August as ‘month 12’ [27]. Each player was assigned a BQ corresponding to their birthdate to create an observed BQ distribution within each of the four cohorts. The observed BQ distributions from each cohort were subsequently compared against the expected BQ distribution calculated from an assumed equal distribution [28]. To examine the likelihood of achieving senior premiership and senior international status once selected at youth levels, further comparisons were provided for the two respective player pathways: (a) premiership pathway and (b) international pathway. As such, the Senior Premiership Player cohort was compared against the expected U18 England Rugby Centre of Excellence Player BQ distribution, while the Senior England Player cohort was compared against the expected U18 England Player BQ distribution.

### 2.3. Data Analysis

Data were analysed using a chi-square (χ^2^) goodness-of-fit test to compare the observed BQ distributions with expected BQ distributions. Since this test does not reveal the magnitude of difference between the BQ distributions for significant χ^2^ outputs, Cramer’s V was also used. The Cramer’s V was interpreted as per conventional thresholds for correlation, whereby a value of 0.06 or more indicated a small effect size, 0.17 or more indicated a medium effect size, and 0.29 or more indicated a large effect size [29]. The odds ratios (ORs) and 95% confidence intervals (CIs; 1 marked no association) were calculated to compare the odds for a player being represented based on their BQ.

## 3. Results

**Figure 1:**
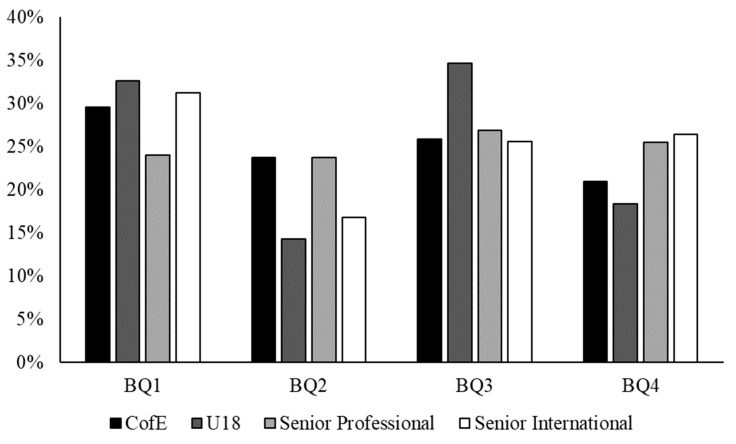
The BQ distributions across each cohort (CoE = U18 England Rugby Centre of Excellence Player; U18 = U18 England Player; Senior Professional = Senior Premiership Player; Senior International = Senior England Player).

Table 1 shows the chi-square analysis of the four cohorts (i.e., U18 England Rugby Centre of Excellence Player, U18 England Player, Senior Premiership Player, and Senior England Player). Figure 1 shows the BQ distributions of the four cohorts. Results showed there were no significant differences between the BQ distributions of all four cohorts when compared against an assumed equal distribution (all *p* < 0.05). Further analysis also showed no significant differences when comparing the Senior Premiership Player cohort against the expected U18 England Rugby Centre of Excellence Player BQ distribution (χ^2^ (df = 3) = 2.998, *p* = 0.391). Moreover, there were no significant differences when comparing the Senior England Player cohort against the expected U18 England Player BQ distribution (χ^2^ (df = 3) = 6.964, *p* = 0.073).

## 4. Discussion

The purpose of this study was to examine RAEs during entry into women’s and girls’ premiership and international rugby union pathways in England, as well as to compare them to their respective senior cohorts. The findings revealed no significant difference in the BQ distributions at either youth or senior levels, as well as no significant differences in the BQ distributions of those who were likely to transition from youth to senior levels. This adds to the small body of literature that has examined RAEs in women’s and girls’ rugby union, whilst it presents an opening to consider the youth-to-senior transition in female sport. These findings are contrary to their male equivalents (e.g., [4,20]) and offer some important methodological and practical considerations for researchers and practitioners, such as developmental differences, sport popularity, and sociocultural norms. Based on these findings, we also warn against a ‘copy and paste’ template from the male provision to ensure that the recent growth of the female rugby union does not fall victim to RAEs in the future.

It is widely acknowledged that growth and maturation influence the biopsychosocial development of girls and boys differently, which has important implications for talent identification and development [30]. As an example, peak height velocity (PHV) takes place at age ~12 years in girls in comparison to age ~14 years in boys, with girls increasing stature at a rate of 8 cm/year and boys at 10 cm/year [31]. However, it should be noted that there is considerable variability in the timing and tempo of PHV, which can vary from an age of approximately 10 to 15 years in girls and from 11 to 16 years in boys, with peak growth rates of 5–11 cm/year and 6–13 cm/year, respectively [31]. Physical features are important contributing factors towards RAEs, particularly in the context of rugby union as they are largely characterised by physical skill [21]. Because girls generally mature earlier than boys, it could potentially explain why no RAEs were observed as they have more time to balance out before adulthood.

Coincidingly, whilst early maturing boys tend to outperform their less mature counterparts on tests of strength, power, speed, agility, and endurance [32,33], research has shown that a similar pattern in girls is not as clear. More specifically, although maturation in females may enhance some aspects of physical performance in rugby union, it may not in others. This could be explained by the natural increases in sex-specific fat mass that girls experience, which can negatively impact certain motor skills involving the movement of body mass that is particularly relevant to rugby union performance [34]. Additionally, research has shown that muscle size is 104% greater in men than boys and only 57% greater in women than girls [35]. Considering that muscle size is a primary factor in the improved capacity to produce force and an important skill required in rugby union, these findings may suggest that sex differences in force production following puberty may not influence these qualities in the same way between the sexes [36]. It is also important to consider how these physiological differences impact technical, tactical, psychological, and social outcomes between the sexes in rugby union; however, there is still a paucity of research exploring these characteristics in the female context [37]. Taken together, although maturity may lead to differences between girls with different levels of maturity within the same chronological age, these differences could be less sensitive to the impact of rugby union performance in girls than in boys.

These current findings are somewhat comparable to the girl’s developmental leagues in Canada and New Zealand, as well as the women’s World Cup rosters displayed in [23]. As such, the level of competition and nationality may not influence female RAEs to the same extent as males. More specifically, whilst there seem to be inconsistencies in men’s rugby union, the preliminary evidence suggests that women’s RAEs may be lower or not present and more consistent. Therefore, the universality of traditional RAEs in women’s and girls’ rugby union needs to be questioned [22]. Indeed, this notion has been previously shown in European football players, whereby males had significant RAEs at U17 and U19 levels but not at senior levels, whereas no RAEs were observed at any level for female players [38]. Due to the discrepancies of RAEs based on competition level and nationality coincided with the much larger evidence base in men’s rugby union, it is important to emphasise that the current findings are only representative of the English context and should not be considered as homogenous until further research is conducted across other female cohorts.

Although findings were not statistically significant, it is important to not overlook the descriptive statistics that show signs of RAEs, which have similar statistically significant BQ percentage distributions in larger cohort studies. In this context, the results of this present study are in contrast to the reversal effects that have been noted in male rugby union (e.g., [9,20]). In fact, there are signs of ‘knock-on effects’ of RAEs, whereby BQ1s are overrepresented at both youth levels and senior levels. Specifically, BQ1s were more likely to be selected into the U18 England Rugby Centre of Excellence (BQ1 = 30% vs. BQ4 = 20%), England U18s (BQ1 = 33% vs. BQ4 = 18%), senior Premiership (BQ1 = 26% vs. BQ4 = 24%), and senior England international (BQ1 = 31% vs. BQ4 = 26%) when compared to BQ4s. This also had a similar trend to boys’ data, whereby higher competition levels (i.e., England U18s) showed greater RAEs compared to lower levels (i.e., U18 England Rugby Centre of Excellence). Additionally, albeit insignificantly, when comparing the Senior Premiership Player cohort against the expected U18 England Rugby Centre of Excellence Player BQ distribution and the Senior England Player cohort against the expected U18 England Player BQ distribution, BQ1s were more likely to transition from youth to senior levels compared to BQ4s, which is contrast to previous data in male studies (e.g., [4,39]). This perhaps raises questions around the age group structures that are implemented into women’s and girls’ sports, as well as the ‘underdog hypothesis’ (or equivalent) as a utility for challenge in this context. However, it is also important to reiterate that these findings were not statistically significant and should continue to be evaluated in larger, longitudinal studies as women’s and girls’ talent pathways continue to develop in England.

Female players make up one-quarter of the worldwide population that participates in rugby union [40]. Compared to these global statistics, however, the English sample in a recent study [11] on age grade players from U7 to U18 showed a greater gender gap in participation between boys (*n* = 228,206; 91%) and girls (*n* = 23,563; 9%). Whilst it is beyond the scope of this study to underscore these participation differences between the sexes, it would be unjust to overlook this factor, particularly as sport popularity is an important mechanism of RAEs in women’s and girls’ sport and could help explain the current findings [27]. Key barriers that minimise or limit participation are perhaps due to the traditional masculine identity of rugby union, the social stigma attached to playing a ‘non-feminine’ sport, or the social stereotypes that surround women rugby union players (e.g., [41,42]). Moreover, it has been well documented that women face poorer career prospects, comprise fewer role models, and are often exposed to inequality throughout sport compared to males [43]. Additionally, despite not being the specific focus of this work, it was previously established that sport popularity may impact the magnitude of RAE effects [44]. For instance, they are more pronounced in popular sports, such as basketball or soccer, than other less popular sports, which combined with the lower participation rate of female players in rugby union may contribute towards the current results. Although these factors may lead to a decreasing level of motivation to participate in rugby union for the next generation of female players, it is also important to consider the continuously growing number of female participants (e.g., a 142% global increase was recorded from 2012 to 2016 [40]). Therefore, whilst it appears that efforts are being made to facilitate more appropriate female competition in rugby union, it is suggested that sociocultural aspects of gender participation require further inquiry.

Organisational structures are responsible for designing and implementing policies in youth sports. In the context of the present sample, England Rugby and World Rugby play crucial roles in how RAEs occur in rugby union since they are responsible for how players are banded during youth competition. Whilst it is somewhat pleasing to see that there are no significant RAEs in this present study, it is also surprising. Although there are likely several reasons why no RAEs were observed, the growing popularity and professionalisation of women’s and girls’ rugby union may be having an impact on the future of RAEs in this cohort. To be specific, this sample analysed retrospective data from senior cohorts as well as snapshots of data from U18 cohorts, both of which are likely products from the pre-existing talent system that was less popular, less professionalised, and less funded. Contrastingly, recent findings across the new girl’s age grade pathway in England revealed significant RAEs across nine out of twelve annual age categories that favoured relatively older players [11]. Since these age groups will form the core of the proceeding youth rosters and senior teams in the years to come, it is plausible to suggest that the female talent system could fall victim to its biennial age group approach. Indeed, this will have important implications for female players and their future sports careers. In an attempt to overcome some of these challenges, many unions are implementing strategies to support women’s and girls’ rugby union.

Women and girls are not men and boys. Therefore, we should not copy and paste male organisational structures for females and expect them to work in the same way. This is the beginning of an exciting journey for women and girls’ rugby union and, therefore, an important and unique opportunity to design and implement modern and innovative policies which help females thrive, regardless of their age. As a result, we should also evaluate new policies and practical strategies to test their effectiveness with the aim of improving over the immediate, short-term, and long-term timescales. Although the intention of using age grouping is to create fairness and equitable competition, it can have a detrimental impact on talent identification and development opportunities. This has been shown across a variety of popular women’s and girls’ sports (see [26] for a review). As such, although RAEs are yet to significantly impact women’s and girls’ talent pathways across rugby union in England, key stakeholders employed in governing bodies who are responsible for policy making are encouraged to explore alternative group banding strategies and look beyond an age grouping approach to create more equitable and effective talent development pathways (see [2]).

Women’s and girls’ rugby union is at a watershed moment. New policies are being designed and implemented as priority is being placed on the development of female talent pathways in England. For instance, there is a newly formed women’s rugby union league in England (i.e., 2020) with a growing number of full-time, professional players, while the U18 England Rugby Centre of Excellence and England U18 programmes have just been restructured (i.e., 2020). In light of the lack of data available within women’s and girls’ pathways, only recently have we been able to collect and analyse statistics in this context for this current study. It is, therefore, important to recognise the many relative age lessons learnt in the male pathway from this current study when designing the emerging female structures to ensure the same issues are not recreated and instead use this as an opportunity to create contemporary organisational structures and more appropriate youth rugby union settings for women and girls [45].

### Limitations and Future Directions

This study is not without its limitations. Although we were able to capture a larger, retrospective sample across both senior cohorts, we only included a single season from the youth samples, resulting in relatively small, isolated examples. Moreover, the high proportion of missing birthdates from the England senior cohort is also noteworthy. Unfortunately, this was due to the dataset that was available; however, the recent England Rugby funding into women’s and girls’ rugby union in England involves more information being collected and stored for the future. Additionally, although findings were insignificant throughout the current study, the girls' descriptive data were comparable to some of the significant results of the higher populated boys’ literature and therefore should be interpreted with caution. Other player characteristics should be considered in future research to ensure we better understand how competition level, playing position, and nationality can influence RAEs in women’s and girls’ rugby union, which is already readily available in the context of men’s and boys’ rugby union.

To help inform their organisational structures in the long term and create a more accurate picture of their player pathways, clubs and governing bodies are encouraged to begin or continue to gather women’s and girls’ data. More specifically, future research is required to explore competition level, performance outcomes, career duration, and playing position based on relative age in order to provide a clearer picture of how women and girls RAEs operate in rugby union. Moreover, priority should be placed on evaluating new policies in women’s and girls’ rugby union to ensure they are meeting their intended objectives (i.e., growing participation and performance), whilst eliminating unintended consequences, such as developing RAEs in their talent pathways. This should coincide with outlining the important developmental and performance differences between sexes so organisational structures in rugby union can meet the needs of female players. Lastly, it is also important to consider other possible selection and development biases that are prevalent throughout rugby union talent pathways, such as relative access to wealth and birthplace effects, which may create a recipe to exacerbate some of the existing RAEs in women’s and girls’ rugby union [46] but remains virtually non-existent in the literature.

## 5. Conclusions

The purpose of this study was to capture the birthdate distribution of the women’s and girls’ professional and international rugby union pathways in England. This was a priority due to the data only recently becoming available, which coincided with the newly professionalized female rugby union pathways in England, and to ensure we are not recreating the same RAEs and issues that have been previously shown in male rugby union. Interestingly, contrary to our hypotheses, results showed no RAEs across all cohorts. Importantly, though, it was highlighted that descriptive statistics showed a skewed birthdate distribution in both U18 England Rugby Centre of Excellence Player (BQ1 = 30% vs. BQ4 = 20%) and U18 England Player cohort (BQ1 = 33% vs. BQ4 = 18%). Therefore, we encourage governing bodies and policymakers to not copy and paste male organisational structures during the creation of new pathways for female rugby union players and instead seek to design, implement, and evaluate new and innovative strategies which help widen participation and the potential pool of talent. Moving forward, it will be vital to evaluate the ongoing transformation of women’s and girls’ rugby union in England (and beyond) to ensure the most appropriate environment is created for every young player to achieve their potential, with research adopting a multidimensional approach to explore the potential obstacles along the way.

## Figures and Tables

**Table 1:** The chi-square analysis across each cohort.

Cohort	BQ1	BQ2	BQ3	BQ4	Total	χ^2^ (df = 3)	*p*	Cramer’s V	OR (BQ1 vs. BQ 4)	95% CI
U18 England Rugby Centre of Excellence Player	96 (30%)	77 (24%)	84 (26%)	68 (20%)	325	5.154	0.161	0.05	1.41	(0.91, 2.19)
U18 England Player	16 (33%)	7 (14%)	17 (35%)	9 (18%)	49	6.102	0.107	0.14	1.78	(0.57, 5.55)
Senior Premiership Player	231 (26%)	213 (25%)	213 (25%)	211 (24%)	868	1.217	0.749	0.02	1.09	(0.83, 1.43)
Senior England Player	39 (31%)	21 (17%)	32 (26%)	33 (26%)	125	5.4	0.145	0.08	1.18	(0.60, 2.32)

## Data Availability

Data is contained within the article.
